# Influence of the skeletal muscle index on pharmacokinetics and toxicity of fluorouracil

**DOI:** 10.1002/cam4.5118

**Published:** 2022-08-08

**Authors:** Eduard Schmulenson, Nigina Zimmermann, Lothar Müller, Stefanie Kapsa, Iryna Sihinevich, Ulrich Jaehde

**Affiliations:** ^1^ Department of Clinical Pharmacy Institute of Pharmacy, University of Bonn Bonn Germany; ^2^ Onkologie UnterEms Leer Germany

**Keywords:** body composition, fluorouracil, pharmacokinetics, skeletal muscle index

## Abstract

**Background:**

The body composition of patients has been associated with tolerability and effectiveness of anticancer therapy. This study aimed to assess the influence of the skeletal muscle index (SMI) on the pharmacokinetics and toxicity of fluorouracil.

**Methods:**

Patients treated in an oncological practice with fluorouracil‐based chemotherapy and undergoing therapeutic drug monitoring were retrospectively investigated. Computed tomography images were analyzed to measure abdominal skeletal muscle areas in Hounsfield units for the psoas major muscle, back and total skeletal muscle to determine the SMI. For the latter, an automated segmentation method was used additionally. SMI measures were tested as covariates on fluorouracil clearance in a population pharmacokinetic model. Furthermore, regression analyses were performed to analyze the influence of SMI measures on the probability of clinically relevant adverse events (CTCAE grades ≥ 2).

**Results:**

Fluorouracil plasma concentrations of 111 patients were available. Covariate analyses showed significant improvements of the model fit by all SMI measures. However, interindividual variability of fluorouracil clearance was only slightly reduced, whereas the SMI of the back muscle showed the largest reduction (−1.1 percentage points). Lower SMI values of the back muscle increased the probability for polyneuropathy and lower SMI of the psoas increased the probability for fatigue.

**Conclusions:**

Our results suggest that pharmacokinetics and toxicity of fluorouracil may be associated with specific SMI measures which deserve further investigation.

## Graphical abstract

In sarcopenic cancer patients, the influence of altered body composition on clinical outcome of anticancer drug therapy is still not completely understood. Results from population pharmacokinetic modeling and logistic regression suggest that skeletal muscle index (SMI) measures may be associated with clearance of fluorouracil (5FU) and the occurrence of fatigue and polyneuropathy, respectively. These relationships deserve further investigation.

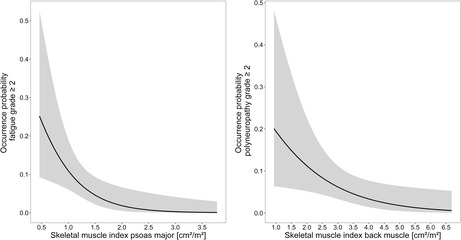

## 1 INTRODUCTION

Fluorouracil (5FU) is still one of the cornerstones for the treatment of various solid tumors, particularly colorectal and head and neck cancer.^1^, ^2^, ^3^ Typically, 5FU is dosed according to the patient's body surface area (BSA), resulting in a wide range of variability in 5FU plasma concentrations.^4^ This pharmacokinetic variability may result individually in an insufficient response to 5FU therapy or intolerable toxicity, leading to treatment discontinuations. In fact, approximately 60% of patients treated with 5FU are reported being underdosed, whereas about 15% being overdosed when BSA‐based dosing is applied.^5^

Due to its hydrophilic nature, the volume of distribution of 5FU is highly correlated with lean body mass (LBM) which includes muscle mass.^6^ Since BSA does not account for changes in body composition, this additional knowledge could be of high interest when sarcopenic cancer patients are treated with anticancer drugs.^7^ Several studies found that sarcopenia in cancer patients is a predictor for low overall survival in various tumors.^8^, ^9^, ^10^, ^11^, ^12^ An association between muscle status and toxicity under therapy with various anticancer drugs was shown as well.^12^ In particular, patients with a dose‐limiting toxicity under 5FU chemotherapy had a higher 5FU dose per kg of LBM compared to patients with a lower grade toxicity.^13^, ^14^ Other metrics of body composition (total body water, fat‐free mass) could be linked to 5FU pharmacokinetics,^15^ whereas a LBM‐normalized 5FU dose was not correlated with 5FU exposure, defined as area under the concentration‐time curve (AUC).^16^

Sarcopenia is usually defined by a reduction in skeletal muscle index (SMI) which is calculated by the total muscle cross‐sectional area at the third lumbar vertebra (L3) normalized to the squared patient's height (cm^2^/m^2^).^17^ So far, the relationship of SMI with 5FU pharmacokinetics has not been evaluated. The aim of this study was therefore to investigate the influence of the SMI on 5FU pharmacokinetics as well as 5FU‐associated toxicity.

## 2 METHODS

### 2.1 Patients and data

In this study, patients under a 5FU‐based, infusional chemotherapy from the oncological outpatient clinic UnterEms in Leer, Germany, were retrospectively analyzed. Patients with documented therapeutic drug monitoring of 5FU, that is, quantification of 5FU plasma concentrations, and at least one computed tomography (CT) scan of the L3 area were included for analysis. 5FU plasma concentrations were obtained at steady state during continuous infusion and quantified using the My5‐FU™ immunoassay (Saladax Biomedical Inc., Bethlehem, PA, USA) with a lower limit of quantification of 86 ng/ml.^18^ Dose adjustments were performed at the discretion of the treating oncologist. Adverse events (AE) were graded at each patient visit according to the Common Terminology Criteria for Adverse Events (CTCAE), version 5.0.^19^ In order to ensure that the muscle status corresponded to measured 5FU plasma concentrations and AE, a maximum time frame between CT scan and blood sampling/AE documentation had to be defined. Chung et al. found a median change in SMI values of 8.7% in patients with stage III or high‐risk stage II colon cancer treated with the FOLFOX scheme (5FU, folinate, oxaliplatin) within 210 days between preoperative and post‐chemotherapy CT.^20^ In addition, the mean measurement error of SMI quantification via CT scans was reported to be 8.5%.^21^ Based on this information, a maximum time frame of ±205 days between CT scan and blood sampling was defined excluding patients with a larger temporal distance between SMI and plasma concentration measurements/AE documentations from the analysis. The study was approved by the ethics committee at the Faculty of Medicine of the University of Bonn (protocol code 014/18).

### 2.2 Image analysis

The SMI was assessed using routinely collected CT images from different radiological practices. As recommended by the European Working Group on Sarcopenia in Older People, measurements were performed at the L3 level since the individual skeletal muscle areas correlated the most with overall skeletal muscle.^22^ Muscle areas were measured based on Hounsfield units (HU) as well as an automated segmentation method provided by the software sliceOmatic® version 5.0 (TomoVision, Magog, Canada).^23^ The HU range was set to the density of skeletal muscle (35–50 HU) to exclude any areas of different tissues.^24^ Using this “Hounsfield method,” the psoas major, back muscle, and total skeletal muscle at the L3 level were examined. For the latter, the automated segmentation method was additionally used. An overview of the different SMI measures and the respective muscle areas is provided in the Supporting Information (SI) 1, Table S1‐1. All SMI measures were obtained by dividing the respective measurements by the squared patient's height.

### 2.3 Population pharmacokinetic analysis

The influence of the different SMI measures on 5FU pharmacokinetics (PK) was analyzed in a population PK model of 5FU. This model was initially developed at the Department of Clinical Pharmacy at the University of Bonn using data from the study of Wilhelm et al.^25^ Modeling was performed with the non‐linear mixed effect modeling software NONMEM® version 7.2^26^ combined with implemented scripts in PsN (version 3.6.2).^27^, ^28^ NONMEM® uses the maximum likelihood method to simultaneously estimate population values of fixed‐effect parameters (e.g., drug clearance) and values of random‐effect variables (e.g., interindividual and residual variability) in order to obtain individual parameters. Model parameters were estimated by the first‐order conditional estimation method with interaction.^26^ The likelihood‐ratio test was used to discriminate between nested models. A nested model was considered superior to another when the objective function value (OFV), provided by NONMEM®, was reduced by 3.84 points (chi‐square value, *p* < 0.05, one degree of freedom). Covariates which are able to explain interindividual variability (IIV) in 5FU clearance and volume of distribution were investigated as well, including age, sex, infusion time (24 or 46 h, coded as a binary covariate), laboratory parameters (creatinine, bilirubin, ALT, AST, GGT, LDH), tumor markers (CA 19–9, CEA), and BSA. These were implemented into the model in a stepwise forward inclusion and backward elimination approach using the *scm* script provided by PsN with an included fixed set of parameter‐covariate parametrizations (linear, piece‐wise linear, exponential, power relations).^27^, ^28^ In the forward inclusion step, covariates which led to a significant decrease of the OFV (*p* < 0.05) were kept for further evaluation. This model was then re‐evaluated by backward elimination of each included covariate with a significance level of *p* < 0.01. If a covariate was still significant in this step, it was eventually kept in the model. Model robustness and precision and bias of parameter estimates were evaluated by a non‐parametric bootstrap analysis without stratification. Median and 95% confidence intervals of parameter estimates were derived from 1000 replicate datasets obtained from sampling individuals from the original dataset with replacement.

The population pharmacokinetic model was then applied to the dataset of this study and revised, where necessary. In this population PK analysis, NONMEM® version 7.5^29^ and PsN (version 5.0.0)^27^, ^28^ were used for model development and R (version 4.1.0)^30^ was used for visualization of results. Piraña (version 2.9.7) served as front interface.^31^ Based on this revised model, a covariate analysis was performed in order to explore if the different SMI measures had an influence on 5FU clearance and volume of distribution. Each SMI measure was individually tested as a covariate on 5FU PK parameters and included if a statistically significant reduction (*p* < 0.05) of the OFV was found. Exponential functions were tested to describe the relationship between the different SMI measures and 5FU PK parameters. The model fit was assessed by goodness‐of‐fit plots^32^ and prediction‐corrected visual predictive checks^33^ based on 1000 dataset simulations. Additionally, a non‐parametric bootstrap analysis without stratification, as described above, was performed.

### 2.4 Logistic regression

The influence of the SMI on 5FU toxicity was evaluated in a logistic regression analysis by correlating SMI measures with AE severity. For this analysis, every AE with a CTCAE grade of 2 or higher was defined as severe and hence clinically relevant. The AE were coded binary with “0” for CTCAE grades 0 and 1 and “1” for grades 2 to 4, respectively. Documented AE included polyneuropathy, stomatitis, hand‐foot syndrome, fatigue, diarrhea, nausea, and emesis. The probability (P) for every AE was calculated as follows:

(1) $$P\left(\mathrm{AE}\geq \text{CTCAE grade}\;2\right)=\frac{1}{1+e^{-z}}\;$$

The logit of z determines the linear regression model of the independent variable and consists of the observed SMI measures ($x_{k}$), the regression coefficients ($\beta_{k}$), and an error term (ε):

(2) $$z=\beta_{0}+\beta_{1}\times x_{1}+\beta_{2}\times x_{2}+\beta_{3}\times x_{3}+\ldots \beta_{k}\times x_{k}+\varepsilon$$

For every performed logistic regression, odds were calculated relating the probability of a severe AE to the probability of non‐occurrence:

(3) $$\text{Odds}=\frac{P\;\left(\text{CTCAE grade}\geq 2\right)}{P\left(\text{CTCAE grade}<2\right)}=\frac{P\;\left(\text{CTCAE grade}\geq 2\right)}{1-P\;\left(\text{CTCAE}\;gr\mathrm{ade}\geq 2\right)}$$

Based on the odds obtained, the relative probability to develop a severe AE when the SMI increases by one unit was assessed as Odds Ratio (OR):

(4) $$\text{OR}=\frac{\text{Odds after}\;1{\mathrm{cm}}^{2}/m^{2}\;\text{increase in}\;\mathrm{SMI}}{\text{Odds before}\;1{\mathrm{cm}}^{2}/m^{2}\;\text{increase in}\;\mathrm{SMI}}$$

## 3 RESULTS

### 3.1 Patient characteristics

The dataset consisted of routinely collected data from 175 patients between September 2014 and July 2020. Twenty of them had to be excluded due to missing CT images. Further 44 patients were excluded because the time frame between their CT scan and blood sampling was longer than 205 days (see “Methods” section). The remaining 111 patients were included for further analyses (Table 1). For the development of the population PK model, 395 5FU plasma concentration measurements were included. All included patients received 24‐h infusions of 5FU.

**TABLE 1:** Patient characteristics

Characteristic	Median (range) or *n*
**Demographics**	
Sex	
Male	75
Female	36
Age (years)	64 (35–84)
Body surface area (m^2^)	1.97 (1.47–2.85)
**Skeletal muscle indices (SMI)**	
SMI psoas major (cm^2^/m^2^)	1.48 (0.46–3.78)
SMI back muscle (cm^2^/m^2^)	3.78 (0.94–8.41)
SMI total muscle (Hounsfield method) (cm^2^/m^2^)	9.58 (4.12–18.82)
SMI total muscle (Segmentation method) (cm^2^/m^2^)	50.26 (25.47–92.67)
**Therapy‐related details**	
5FU dose (mg/m^2^)	2283 (1441–3641)
5FU AUC^a^ (mg × h/L)	19.7 (2.1–45.0)
Number of observed cycles per patient	2 (1–5)
Therapy regimen	
AIO^b^	26
FUFOX^c^ (including monoclonal antibodies)	22
Paclitaxel/cisplatin/5FU/folinate	30
Other	32
**Tumor entity**	
Colorectal cancer	56
Gastroesophageal cancer	33
Pancreatic cancer	12
Other	10

^a^ Calculated by multiplying the infusion time with the measured steady‐state concentration.

^b^ Weekly 5FU infusion (2600 mg/m^2^) over 24 h in combination with folinate (500 mg/m^2^).

^c^ Weekly 5FU infusion (2000 mg/m^2^) over 24 h in combination with folinate (500 mg/m^2^) and oxaliplatin (50 mg/m^2^).

### 3.2 Influence of the skeletal muscle index on 5FU pharmacokinetics

First, an initial population PK model based on data from the study of Wilhelm et al.^25^ was developed. A one‐compartment model with linear elimination turned out to be the best model to describe 5FU disposition. IIV terms were implemented on 5FU clearance, volume of distribution, and residual variability. The latter consisted of an additive as well as a proportional term.^34^ However, due to model instabilities, estimates of the volume of distribution and its IIV had to be fixed to previously estimated values. Based on analyses of rich PK data from our group and data from the Cantonal Hospital St. Gallen, Switzerland, volume of distribution and its IIV were fixed to 46.1 L and 51.1%, respectively.^35^, ^36^, ^37^ Therefore, covariates could only be tested on 5FU clearance. After the forward inclusion step of the covariate analysis, BSA, infusion time, and LDH concentration were found to be significant linear covariates on 5FU clearance. LDH concentration was excluded after performing the backward elimination step. Ultimately, BSA and infusion time remained in the model. However, the bootstrap analysis revealed the estimate of the infusion time effect to be unreliable since its 95% confidence intervals included zero. Hence, this parameter was excluded from the final model. In Table 2, the development steps of the population PK model are shown. Table 3 depicts the final population PK parameter estimates as well as the median values of the bootstrap analysis.

**TABLE 2:** Development steps and covariate analysis of the initial 5FU population pharmacokinetic model based on data from Wilhelm et al.^25^

Model number	Description	OFV	∆OFV	*p* value
1	**Base model** One compartment with linear elimination Fixed values for volume of distribution and its IIV Combined additive and proportional residual error model	−611.310	0	—
2	**Covariate model after forward inclusion step** Inclusion of BSA, infusion time, and LDH concentration on 5FU clearance	−653.107	−41.797	<0.0001^a^
3	**Covariate model after backward elimination step** Exclusion of LDH concentration as covariate	−647.132	+5.975	0.0145
4	**Final model** Exclusion of infusion time as covariate after bootstrap analysis	−637.090	+10.042	0.0064

Abbreviations: ∆OFV, Difference in objective function value; BSA, Body surface area; IIV, Interindividual variability; OFV, Objective function value.

^a^ Three degrees of freedom.

**TABLE 3:** Parameter estimates of the initial 5FU population pharmacokinetic model based on data from Wilhelm et al.^25^

Parameter	Estimate	Bootstrap median (95% confidence intervals)
CL_5FU_ [L/h]	209	209 (198–220)
V_5FU_ [L]	46.1 (fixed)	46.1 (fixed)
BSA effect on CL_5FU_	0.681	0.688 (0.455–0.933)
*Interindividual variability*		
CL_5FU_ [%CV]	16.3	15.5 (10.3–19.2)
V_5FU_ [%CV]	51.1 (fixed)	51.1 (fixed)
Residual variability [%CV]	37.5	36.1 (18.7–52.2)
*Residual variability*		
Additional error term [ng/ml]	77.4	87.2 (54.7–139)
Proportional error term [%]	19.8	18.8 (14.7–23.6)

Abbreviations: BSA, body surface area; CL, clearance; CV, coefficient of variation; V, volume of distribution.

The developed population PK model was applied to the dataset of this study. Running this initial model revealed that the estimate of the additive term of the residual variability ran into a boundary close to zero. In addition, the IIV term on residual variability was estimated with a relative standard error (RSE) of 86% and shrinkage^38^ of 76%. Both parameters were assumed to be negligible and thus removed from the model leading to a non‐significant increase in OFV (Table 4).

**TABLE 4:** Model development steps and covariate analysis of the revised model

Model number	Description	OFV	∆OFV	*p* value	IIV Clearance [%CV]
1	Initial model without BSA as covariate (Table 3)	−754.341	0	—	26.6
2	Initial model with BSA as covariate (Table 3)	−792.218	−37.877	<0.00001	22.0
3	Initial model with BSA as covariate (Table 3), without additive term and IIV term of residual variability (**Revised model**)	−791.624	+0.594	(0.74^a^, referring to model 2)	22.0
3a	Revised model + SMI total muscle (Hounsfield method)	−798.302	−6.678	0.0098	21.5
3b	Revised model + SMI total muscle (segmentation method)	−798.408	−6.784	0.0092	21.1
3c	Revised model + SMI psoas major	−799.070	−7.446	0.0064	21.1
3d	Revised model + SMI back muscle	−803.910	−12.286	0.00046	20.9

Abbreviations: ∆OFV, Difference in objective function value; BSA, Body surface area; CV, Coefficient of variation; IIV, Interindividual variability; OFV, Objective function value; SMI, Skeletal muscle index.

^a^ Two degrees of freedom.

This revised model was then used for covariate analysis in which the different SMI measures were individually tested on 5FU clearance. Its results are presented in Table 4. All four SMI parameters showed a statistically significant reduction of OFV when included in the model. Inclusion of the SMI of the back muscle led to the largest OFV drop along with the highest reduction in IIV of 5FU clearance (−1.1 percentage points). Therefore, this covariate was chosen for the final model. 5FU clearance was parametrized as follows (Equation 5):
(5)

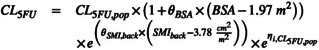

 Here, CL_5FU_ denotes for the individual 5FU clearance estimate, CL_5FU,pop_ for the population estimate of 5FU clearance, θ_BSA_ is the covariate effect estimate of the body surface area, BSA is the observed body surface area, θ_SMI,back_ denotes for the covariate effect estimate of the SMI of the back muscle, SMI_back_ for the observed SMI of the back muscle, and η_i,CL5FU,pop_ represents the IIV term for the 5FU population clearance of the ith individual with a mean of 0 and a variance of ω^2^. Both covariate effects were centered around the respective observed median values of the study population.

**FIGURE 1:**
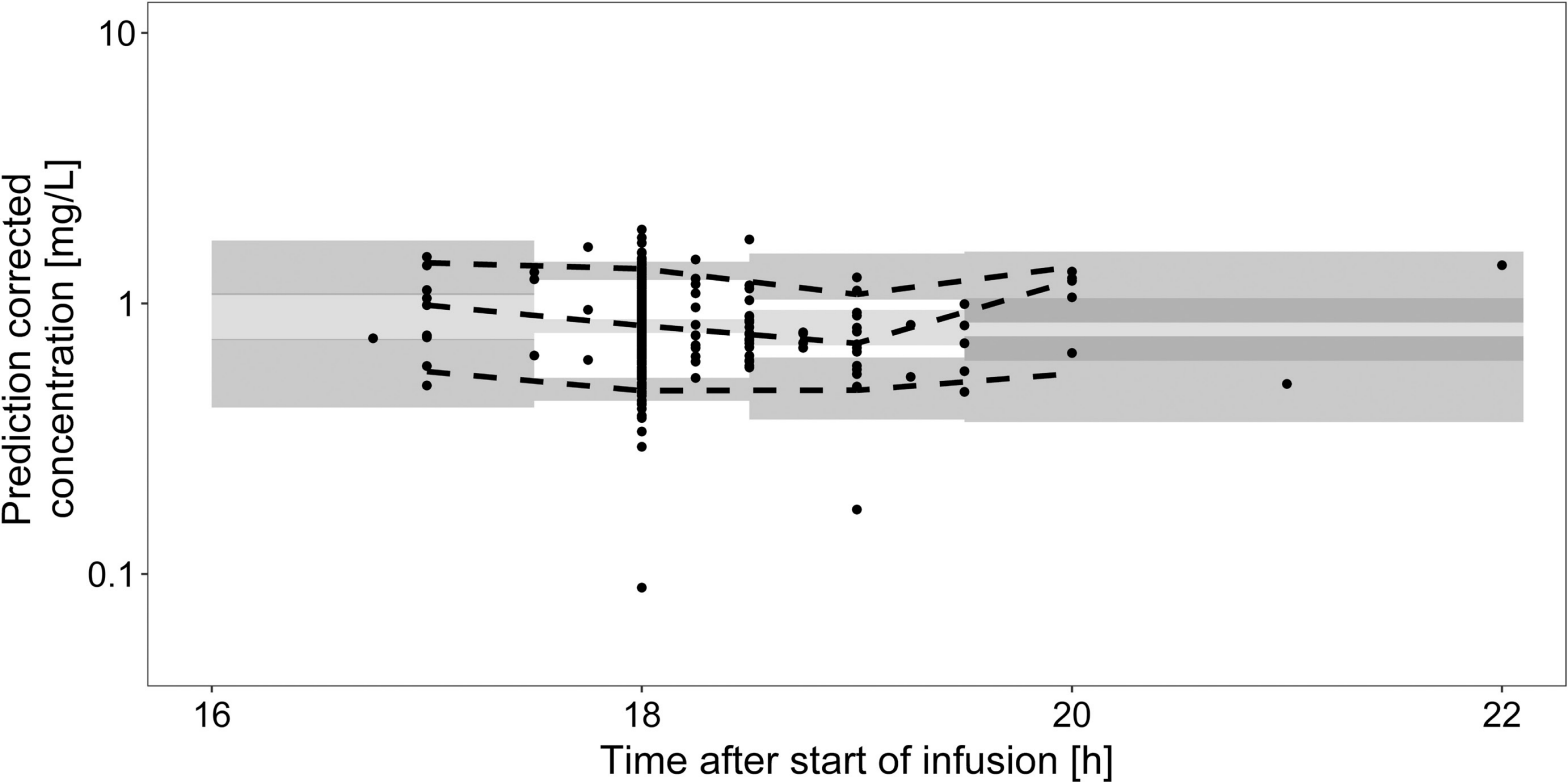
Prediction‐corrected visual predictive check of the final population pharmacokinetic model of 5FU. Black dots: Prediction‐corrected observations, dashed lines: 90% interval and median of the prediction‐corrected observations, dark gray shaded area: 95% confidence intervals of the 5th and 95th prediction interval, light gray shaded area: 95% confidence interval of the predicted median.

The prediction‐corrected visual predictive check (Figure 1) as well as goodness‐of‐fit plots (see SI 2, Figure S2‐1) of the final model showed a reasonable model fit. Final parameter estimates along with bootstrap results are presented in Table 5. The NONMEM® code of the final model is outlined in SI 3.

**TABLE 5:** Parameter and bootstrap estimates of the final model

Parameter	Estimate (relative standard error, %)	Shrinkage [%]	Bootstrap median (95% confidence intervals)
CL_5FU_ [L/h]	223 (2.4)		223 (212–234)
V_5FU_ [L]	46.1 (fixed estimate)		46.1 (fixed estimate)
BSA effect on CL_5FU_	0.794 (14.5)		0.796 (0.543–1.02)
SMI_Back_ effect on CL_5FU_	0.0570 (29.8)		0.0575 (0.0283–0.0885)
*Interindividual variability*			
CL_5FU_ [%CV]	20.9 (7.4)	15.1	20.4 (15.8–24.5)
V_5FU_ [%CV]	51.1 (fixed estimate)	100	51.1 (fixed estimate)
*Residual variability*			
Proportional error [%]	21.4 (3.3)	10.6	21.4 (19.0–23.8)

Abbreviations: BSA, body surface area; CL, clearance; CV, coefficient of variation; SMI_back_, skeletal back muscle index; V, volume of distribution.

### 3.3 Influence of skeletal muscle indices on toxicity under 5FU therapy

**FIGURE 2:**
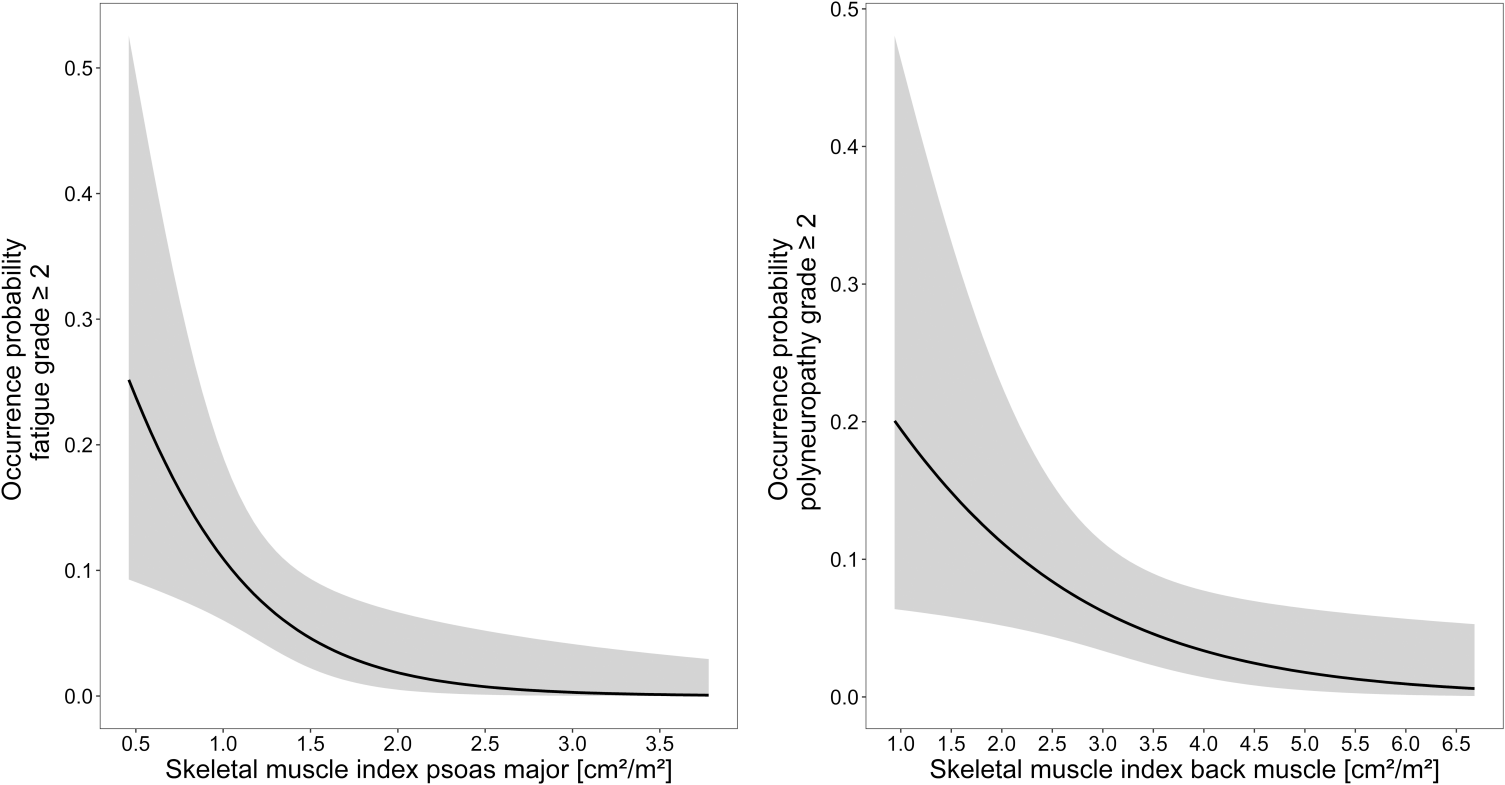
Probability of fatigue and polyneuropathy grade ≥ 2 versus different skeletal muscle indices. Black solid lines are the mean probabilities and the gray shaded areas are the respective 95% confidence intervals.

For every AE, the number of patients suffering from AE grade 2 or higher is presented in SI 4, Table S4‐1. The logistic regression analysis showed statistically significant correlations for two AE. The SMI of the psoas major was significantly correlated to the fatigue syndrome as well as the SMI of the back muscle to the occurrence of clinically relevant polyneuropathy. An increase of the respective SMI by 1 cm^2^/m^2^ decreased the probability of developing the identified AE ≥ grade 2 by 85% and 48%, respectively. The final logistic regression analyses are depicted in Figure 2. Presentations of the odds ratios for all investigated AE are outlined in SI 4, Table S4‐2.

## 4 DISCUSSION

This is the first study evaluating the influence of different SMI measures on 5FU PK and toxicity. The results suggest that selected SMI measures may be associated with 5FU PK. Interpreting the individual SMI measures, it should be kept in mind that SMI parameter values which were obtained by the Hounsfield method were quantified using a range of 35–50 HU. This range differed from HU ranges of other studies^13^, ^14^, ^39^ and resulted in lower absolute values when, for example, comparing the SMI of the total muscle obtained by the Hounsfield method to the same SMI quantified by the segmentation method. However, the observed trends and associations were the same for all SMI measures analyzed in this study. Provided that these findings are confirmed in future prospective studies, the question remains how these findings may be transposed in real‐world patients. In clinical routine, CT imaging of cancer patients is frequently performed, for example, for tumor staging. Therefore, even though muscle status assessment by CT imaging is not routinely conducted, there would already be raw data available to quantify different SMI measures.^40^

Our population PK model was able to adequately describe the observed routine data. However, the sole availability of steady‐state concentrations impeded model development. The estimate of 5FU volume of distribution had to be therefore fixed and could not be used for covariate analysis. Gusella et al. found a significant relationship between total body water and 5FU volume of distribution as well as fat‐free mass and 5FU volume of distribution.^15^ The initially developed 5FU population PK model, which was based on data from Wilhelm et al.,^25^ included BSA as the only significant covariate on 5FU clearance. In general, the identified covariates on 5FU PK vary considerably. A similar influence of BSA was identified in two other published population PK models as well,^41^, ^42^ whereas other models revealed significant effects of sex,^36^, ^43^ age,^44^ or body weight.^45^ When applying the initial model on the new dataset, all SMI measures were positively associated with 5FU clearance when adding them as covariates and significantly improved the model fit. The inclusion of the SMI of the back muscle led to the largest improvement which deserves further investigation on physiological plausibility. While this finding gives additional hints that body composition may influence the PK of 5FU and other anticancer drugs,^15^, ^46^, ^47^, ^48^ the minor reduction of IIV of 5FU clearance revealed that the usage of SMI for dose adjustment purposes may be of limited value. In fact, the inclusion of BSA had a higher impact on 5FU clearance (Table 4). The wide range of SMI measures (Table 1) may further explain the limited reduction of variability in clearance. Interestingly, Molenaar‐Kuijsten et al. could not identify any relationships between the SMI‐derived skeletal muscle mass and the 5FU elimination rate constant in 151 patients treated with its oral prodrug capecitabine.^39^ Williams et al. investigated the influence of LBM which was derived from the SMI on 5FU AUC but could not find any significant differences between sarcopenic and non‐sarcopenic patients.^16^ However, they investigated a smaller patient cohort (25 patients) and only used first cycle plasma concentrations of 5FU, whereas in our study, plasma concentrations from different cycles were available.

As a limitation of our study, it should be noted that the genotypes and the activity of the main metabolizing enzyme, dihydropyrimidine dehydrogenase (DPD), which are important predictors for 5FU exposure^49^, ^50^ were not available for our patient cohort. However, IIV of 5FU clearance was already lower than the average IIV of 40% as reported in an extensive review on 5FU therapeutic drug monitoring by Beumer et al.^5^ This could be explained by the comparatively low prevalence of DPD risk variants which are associated with alleles producing DPD with minimal/no activity (DPYD*2A or DPYD*13) or with alleles producing DPD with decreased metabolic function (c.2846A > T or c.1129–5923C > G). Up to 1% of the European population are carriers of the non‐active variants, whereas the two latter variants are present in about 5% of Europeans.^51^ Keeping in mind the number of patients used for model development (*n* = 75 in the study of Wilhelm et al.,^25^ *n* = 111 in the present study), only few patients presumably were carriers of these DPD risk variants that are associated with a substantial influence on the variability in 5FU clearance.

The findings of the logistic regression analysis showed several significant associations between specific SMI measures and adverse events under 5FU therapy which was generally in accordance with previous studies.^13^, ^14^, ^16^ Williams et al. found a significant positive association of LBM and oxaliplatin clearance and LBM and volume of distribution as well as a significant association of low LBM and high‐grade toxicity in older patients with colorectal cancer. Similar results were identified for cisplatin even though dose‐limiting toxicity was not significantly correlated with body composition.^53^ Our finding of a higher probability of the occurrence of clinically relevant polyneuropathy with decreasing SMI of the back muscle deserves further investigation. Since the psoas muscle is necessary for everyday movement, the reduction of its SMI may be an explanation for the identified increase in the probability of clinically relevant fatigue. It should be noted, however, that our analysis was focused on evaluating the general susceptibility of experiencing clinically relevant AE depending on body composition in patients treated with a 5FU‐based chemotherapy. Regarding concomitant chemotherapy, only qualitative information of its administration was available for our study. In a future study, it would be of high interest to investigate the influence of individual drug PK on AE development in order to distinguish between the respective individual contributions of these drugs. In addition, the patient's performance status should be included in such an analysis as it was reported to be significantly associated with SMI^54^ and a significant predictor for drug toxicity under 5FU‐based chemotherapy.^55^

In conclusion, this retrospective study gives first hints that the SMI as a measure of body composition may be associated with the pharmacokinetic disposition and the development of toxicity of 5FU. A prospective study in which SMI measures and 5FU pharmacokinetics are investigated should provide additional insights into these relevant relationships between body composition and clinical outcome of 5FU‐based chemotherapy.

## AUTHOR CONTRIBUTION

Designed research: ES, NZ, LM, UJ. Performed research: ES, NZ, LM. Analyzed data: ES, NZ, SK, IS, UJ. Wrote or contributed to the writing of the manuscript: ES, NZ, LM, SK, IS, UJ.

## FUNDING INFORMATION

This study was not funded.

## CONFLICT OF INTEREST

All authors declare that they have no conflicts of interest that are relevant to the content of this manuscript.

## ETHICS STATEMENT

This retrospective study was approved by the local medical ethics committee.

## CLINICAL TRIAL REGISTRATION

Due to the retrospective nature of this study, trial registration was not conducted.

## Supporting information


Appendix

## Data Availability

Data are available from the corresponding author upon reasonable request.

## References

[1] van Cutsem E , Cervantes A , Nordlinger B , Arnold D , ESMO Guidelines Working Group . Metastatic colorectal cancer: ESMO clinical practice guidelines for diagnosis, treatment and follow‐up. Ann Oncol. 2014;25(Suppl 3):iii1‐iii9. 25190710 10.1093/annonc/mdu260

[2] Argilés G , Tabernero J , Labianca R , et al. Localised colon cancer: ESMO clinical practice guidelines for diagnosis, treatment and follow‐up. Ann Oncol. 2020;31:1291‐1305. 32702383 10.1016/j.annonc.2020.06.022

[3] Pfister DG , Spencer S , Adelstein D , et al. Head and neck cancers, version 2.2020, NCCN clinical practice guidelines in oncology. J Natl Compr Canc Netw. 2020;18:873‐898. 32634781 10.6004/jnccn.2020.0031

[4] Schmulenson E , Zimmermann N , Mikus G , Joerger M , Jaehde U . Current status and future outlooks on therapeutic drug monitoring of fluorouracil. Expert Opin Drug Metab Toxicol. 2021;17:1407‐1422. 35029518 10.1080/17425255.2021.2029403

[5] Beumer JH , Chu E , Allegra C , et al. Therapeutic drug monitoring in oncology: International Association of Therapeutic Drug Monitoring and Clinical Toxicology Recommendations for 5‐fluorouracil therapy. Clin Pharmacol Ther. 2019;105:598‐613. 29923599 10.1002/cpt.1124 PMC6309286

[6] Morgan DJ , Bray KM . Lean body mass as a predictor of drug dosage. Clin Pharmacokinet. 1994;26:292‐307. 8013162 10.2165/00003088-199426040-00005

[7] Prado CMM , Maia YLM , Ormsbee M , Sawyer M , Baracos V . Assessment of nutritional status in cancer—the relationship between body composition and pharmacokinetics. Anticancer Agents Med Chem. 2013;13:1197‐1203. 23919745 10.2174/18715206113139990322

[8] Martin L , Birdsell L , Macdonald N , et al. Cancer cachexia in the age of obesity: skeletal muscle depletion is a powerful prognostic factor, independent of body mass index. J Clin Oncol. 2013;31:1539‐1547. 23530101 10.1200/JCO.2012.45.2722

[9] Caan BJ , Cespedes Feliciano EM , Prado CM , et al. Association of muscle and adiposity measured by computed tomography with survival in patients with nonmetastatic breast cancer. JAMA Oncol. 2018;4:798‐804. 29621380 10.1001/jamaoncol.2018.0137 PMC6584322

[10] Prado CMM , Lieffers JR , McCargar LJ , et al. Prevalence and clinical implications of sarcopenic obesity in patients with solid tumours of the respiratory and gastrointestinal tracts: a population‐based study. Lancet Oncol. 2008;9:629‐635. 18539529 10.1016/S1470-2045(08)70153-0

[11] Shachar SS , Williams GR , Muss HB , Nishijima TF . Prognostic value of sarcopenia in adults with solid tumours: a meta‐analysis and systematic review. Eur J Cancer. 2016;57:58‐67. 26882087 10.1016/j.ejca.2015.12.030

[12] Ryan AM , Prado CM , Sullivan ES , Power DG , Daly LE . Effects of weight loss and sarcopenia on response to chemotherapy, quality of life, and survival. Nutrition. 2019;67‐68:110539. 10.1016/j.nut.2019.06.020 31522087

[13] Prado CMM , Baracos VE , McCargar LJ , et al. Body composition as an independent determinant of 5‐fluorouracil‐based chemotherapy toxicity. Clin Cancer Res. 2007;13:3264‐3268. 17545532 10.1158/1078-0432.CCR-06-3067

[14] Ali R , Baracos VE , Sawyer MB , et al. Lean body mass as an independent determinant of dose‐limiting toxicity and neuropathy in patients with colon cancer treated with FOLFOX regimens. Cancer Med. 2016;5:607‐616. 26814378 10.1002/cam4.621 PMC4831278

[15] Gusella M , Toso S , Ferrazzi E , Ferrari M , Padrini R . Relationships between body composition parameters and fluorouracil pharmacokinetics. Br J Clin Pharmacol. 2002;54:131‐139. 12207632 10.1046/j.1365-2125.2002.01598.x PMC1874401

[16] Williams GR , Deal AM , Shachar SS , et al. The impact of skeletal muscle on the pharmacokinetics and toxicity of 5‐fluorouracil in colorectal cancer. Cancer Chemother Pharmacol. 2018;81:413‐417. 29159476 10.1007/s00280-017-3487-2 PMC5777882

[17] Baracos VE , Arribas L . Sarcopenic obesity: hidden muscle wasting and its impact for survival and complications of cancer therapy. Ann Oncol. 2018;29:ii1‐ii9. 10.1093/annonc/mdx810 29506228

[18] Beumer JH , Boisdron‐Celle M , Clarke W , et al. Multicenter evaluation of a novel nanoparticle immunoassay for 5‐fluorouracil on the Olympus AU400 analyzer. Ther Drug Monit. 2009;31:688‐694. 19935361 10.1519/JSC.0b013e3181b866d0

[19] National Cancer Institute . Common Terminology Criteria for Adverse Events v5.0 (CTCAE); 2018. Available at: http://ctep.cancer.gov/. Accessed 6 Mar 2022.

[20] Chung E , Lee HS , Cho E‐S , et al. Changes in body composition during adjuvant FOLFOX chemotherapy and overall survival in non‐metastatic colon cancer. Cancers (Basel). 2019;12:60. 31878325 10.3390/cancers12010060 PMC7016804

[21] Mitsiopoulos N , Baumgartner RN , Heymsfield SB , Lyons W , Gallagher D , Ross R . Cadaver validation of skeletal muscle measurement by magnetic resonance imaging and computerized tomography. J Appl Physiol. 1985;1998(85):115‐122. 10.1152/jappl.1998.85.1.115 9655763

[22] Cruz‐Jentoft AJ , Bahat G , Bauer J , et al. Sarcopenia: revised European consensus on definition and diagnosis. Age Ageing. 2019;48:16‐31. 30312372 10.1093/ageing/afy169 PMC6322506

[23] Popuri K , Cobzas D , Esfandiari N , Baracos V , Jagersand M . Body composition assessment in axial CT images using FEM‐based automatic segmentation of skeletal muscle. IEEE Trans Med Imaging. 2016;35:512‐520. 26415164 10.1109/TMI.2015.2479252

[24] Mei K , Schwaiger BJ , Kopp FK , et al. Bone mineral density measurements in vertebral specimens and phantoms using dual‐layer spectral computed tomography. Sci Rep. 2017;7:17519. 29235542 10.1038/s41598-017-17855-4 PMC5727524

[25] Wilhelm M , Mueller L , Miller MC , et al. Prospective, multicenter study of 5‐fluorouracil therapeutic drug monitoring in metastatic colorectal cancer treated in routine clinical practice. Clin Colorectal Cancer. 2016;15:381‐388. 27256667 10.1016/j.clcc.2016.04.001

[26] Beal S , Sheiner LB , Boeckmann A , Bauer RJ . NONMEM User's Guides (1989–2018). Icon Development Solutions; 2018.

[27] Lindbom L , Ribbing J , Jonsson EN . Perl‐speaks‐NONMEM (PsN)—a Perl module for NONMEM related programming. Comput Methods Programs Biomed. 2004;75:85‐94. 15212851 10.1016/j.cmpb.2003.11.003

[28] Lindbom L , Pihlgren P , Jonsson EN , et al. PsN‐toolkit—a collection of computer intensive statistical methods for non‐linear mixed effect modeling using NONMEM. Comput Methods Programs Biomed. 2005;79:241‐257. 16023764 10.1016/j.cmpb.2005.04.005

[29] Bauer RJ . NONMEM Users Guide. Icon Development Solutions; 2021.

[30] R Core Team . (2022). R: a language and environment for statistical computing. R Foundation for Statistical Computing. Available at: http://www.R‐project.org/. Accessed 6 Mar 2022.

[31] Keizer RJ , Karlsson MO , Hooker A . Modeling and simulation workbench for NONMEM: tutorial on Pirana, PsN, and Xpose. CPT Pharmacometrics Syst Pharmacol. 2013;2:e50. 23836189 10.1038/psp.2013.24 PMC3697037

[32] Nguyen THT , Mouksassi M‐S , Holford N , et al. Model evaluation of continuous data Pharmacometric models: metrics and graphics. CPT Pharmacometrics Syst Pharmacol. 2017;6:87‐109. 27884052 10.1002/psp4.12161 PMC5321813

[33] Bergstrand M , Hooker AC , Wallin JE , Karlsson MO . Prediction‐corrected visual predictive checks for diagnosing nonlinear mixed‐effects models. AAPS J. 2011;13:143‐151. 21302010 10.1208/s12248-011-9255-z PMC3085712

[34] Mould DR , Upton RN . Basic concepts in population modeling, simulation, and model‐based drug development‐part 2: introduction to pharmacokinetic modeling methods. CPT Pharmacometrics Syst Pharmacol. 2013;2:e38. 23887688 10.1038/psp.2013.14 PMC3636497

[35] Streit M , Jaehde U , Stremetzne S , et al. Five‐day continuous infusion of 5‐fluorouracil and pulsed folinic acid in patients with metastatic colorectal carcinoma: an effective second‐line regimen. Ann Oncol. 1997;8:163‐165. 10.1023/a:1008257603687 9426339

[36] Mueller F , Büchel B , Köberle D , et al. Gender‐specific elimination of continuous‐infusional 5‐fluorouracil in patients with gastrointestinal malignancies: results from a prospective population pharmacokinetic study. Cancer Chemother Pharmacol. 2013;71:361‐370. 23139054 10.1007/s00280-012-2018-4

[37] Hendrayana T , Kurth V , Krolop L , et al. Variability in fluorouracil exposure during continuous intravenous infusion. Int J Clin Pharmacol Ther. 2012;50:82‐84. 22192656 10.5414/cpp50082

[38] Savic RM , Karlsson MO . Importance of shrinkage in empirical Bayes estimates for diagnostics: problems and solutions. AAPS J. 2009;11:558‐569. 19649712 10.1208/s12248-009-9133-0 PMC2758126

[39] Molenaar‐Kuijsten L , Jacobs BAW , Kurk SA , et al. Worse capecitabine treatment outcome in patients with a low skeletal muscle mass is not explained by altered pharmacokinetics. Cancer Med. 2021;10:4781‐4789. 34121365 10.1002/cam4.4038 PMC8290233

[40] Rinninella E , Cintoni M , Raoul P , et al. Muscle mass, assessed at diagnosis by L3‐CT scan as a prognostic marker of clinical outcomes in patients with gastric cancer: a systematic review and meta‐analysis. Clin Nutr. 2020;39:2045‐2054. 31718876 10.1016/j.clnu.2019.10.021

[41] Arshad U , Ploylearmsaeng S‐A , Karlsson MO , et al. Prediction of exposure‐driven myelotoxicity of continuous infusion 5‐fluorouracil by a semi‐physiological pharmacokinetic‐pharmacodynamic model in gastrointestinal cancer patients. Cancer Chemother Pharmacol. 2020;85:711‐722. 32152679 10.1007/s00280-019-04028-5 PMC7125253

[42] Terret C , Erdociain E , Guimbaud R , et al. Dose and time dependencies of 5‐fluorouracil pharmacokinetics. Clin Pharmacol Ther. 2000;68:270‐279. 11014408 10.1067/mcp.2000.109352

[43] Bressolle F , Joulia JM , Pinguet F , et al. Circadian rhythm of 5‐fluorouracil population pharmacokinetics in patients with metastatic colorectal cancer. Cancer Chemother Pharmacol. 1999;44:295‐302. 10447576 10.1007/s002800050980

[44] Etienne M‐C , Chatelut E , Pivot X , et al. Co‐variables influencing 5‐fluorouracil clearance during continuous venous infusion.A NONMEM analysis. Eur J Cancer. 1998;34:92‐97. 9624244 10.1016/s0959-8049(97)00345-6

[45] Climente‐Martí M , Merino‐Sanjuán M , Almenar‐Cubells D , Jiménez‐Torres NV . A Bayesian method for predicting 5‐fluorouracil pharmacokinetic parameters following short‐term infusion in patients with colorectal cancer. J Pharm Sci. 2003;92:1155‐1165. 12761805 10.1002/jps.10374

[46] Mir O , Coriat R , Blanchet B , et al. Sarcopenia predicts early dose‐limiting toxicities and pharmacokinetics of sorafenib in patients with hepatocellular carcinoma. PLoS ONE. 2012;7:e37563. 22666367 10.1371/journal.pone.0037563 PMC3364283

[47] Prado CMM , Lima ISF , Baracos VE , et al. An exploratory study of body composition as a determinant of epirubicin pharmacokinetics and toxicity. Cancer Chemother Pharmacol. 2011;67:93‐101. 20204364 10.1007/s00280-010-1288-y

[48] Massicotte M‐H , Borget I , Broutin S , et al. Body composition variation and impact of low skeletal muscle mass in patients with advanced medullary thyroid carcinoma treated with vandetanib: results from a placebo‐controlled study. J Clin Endocrinol Metab. 2013;98:2401‐2408. 23543666 10.1210/jc.2013-1115

[49] Lee JJ , Beumer JH , Chu E . Therapeutic drug monitoring of 5‐fluorouracil. Cancer Chemother Pharmacol. 2016;78:447‐464. 27217046 10.1007/s00280-016-3054-2 PMC5204259

[50] Diasio RB , Johnson MR . Dihydropyrimidine dehydrogenase: its role in 5‐fluorouracil clinical toxicity and tumor resistance. Clin Cancer Res. 1999;5:2672‐2673. 10537327

[51] Hamzic S , Aebi S , Joerger M , et al. Fluoropyrimidine chemotherapy: recommendations for DPYD genotyping and therapeutic drug monitoring of the swiss Group of Pharmacogenomics and Personalised Therapy. Swiss Med Wkly. 2020;150:w20375. 33232506 10.4414/smw.2020.20375

[52] Williams GR , Al‐Obaidi M , Rower J , et al. Does oxaliplatin pharmacokinetics (PKs) explain associations between body composition and chemotherapy toxicity risk in older adults with gastrointestinal (GI) cancers? J Clin Oncol. 2021;39:3095.

[53] Chargi N , Molenaar‐Kuijsten L , Huiskamp LFJ , Devriese LA , de Bree R , Huitema ADR . The association of cisplatin pharmacokinetics and skeletal muscle mass in patients with head and neck cancer: the prospective PLATISMA study. Eur J Cancer. 2022;160:92‐99. 34810046 10.1016/j.ejca.2021.10.010

[54] Dolan RD , Daly LE , Simmons CP , et al. The relationship between ECOG‐PS, mGPS, BMI/WL grade and body composition and physical function in patients with advanced cancer. Cancers (Basel). 2020;12:1187. 32397102 10.3390/cancers12051187 PMC7281405

[55] Sargent DJ , Köhne CH , Sanoff HK , et al. Pooled safety and efficacy analysis examining the effect of performance status on outcomes in nine first‐line treatment trials using individual data from patients with metastatic colorectal cancer. J Clin Oncol. 2009;27:1948‐1955. 19255311 10.1200/JCO.2008.20.2879 PMC2669760

