# Supplementary material for: Influence of the skeletal muscle index on pharmacokinetics and toxicity of fluorouracil
Source: Cancer Med. 2022 Aug 8;12(3):2580–9. doi: 10.1002/cam4.5118 (PMC9939223; doi:10.1002/cam4.5118)
Supplement: Supplementary file 1 — Appendix [file CAM4-12-2580-s001.zip › CAM4_5118_S1_Image_Analysis_SuppInfo.docx]

**Image analysis**

Tab. S1-1 Measured skeletal muscle areas and methods used

| **Skeletal muscle index (SMI)** | **Measured skeletal muscle areas** | **Method** |
| --- | --- | --- |
| SMI Psoas | Psoas major | Hounsfield |
| SMI back muscle | Erector spinae  Quadratus lumborum | Hounsfield |
| SMI total skeletal muscle | Psoas major  Erector spinae  Quadratus lumborum  Transversus abdominalis  Internal/external Musculus obliquus abdominis  Rectus abdominis | Hounsfield/Segmentation |
