# Supplementary material for: Influence of the skeletal muscle index on pharmacokinetics and toxicity of fluorouracil
Source: Cancer Med. 2022 Aug 8;12(3):2580–9. doi: 10.1002/cam4.5118 (PMC9939223; doi:10.1002/cam4.5118)
Supplement: Supplementary file 1 — Appendix [file CAM4-12-2580-s001.zip › CAM4_5118_S2_Population_Pharmacokinetic_Analysis_SuppInfo.docx]

**Population pharmacokinetic analysis**


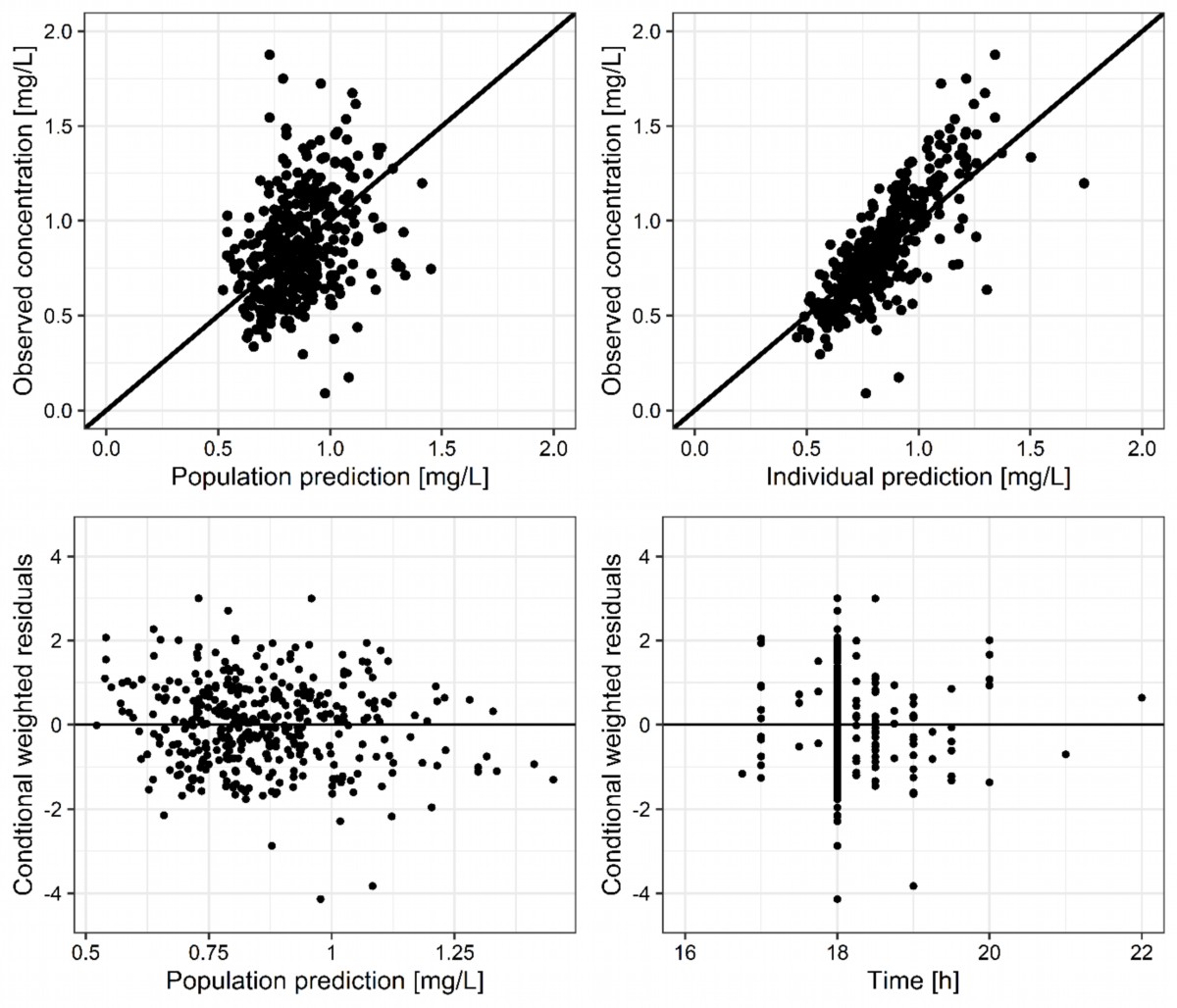


Fig. S2 Goodness-of-fit plots for model-predicted fluorouracil concentrations. The black lines indicate the lines of identity.
