# Supplementary material for: Influence of the skeletal muscle index on pharmacokinetics and toxicity of fluorouracil
Source: Cancer Med. 2022 Aug 8;12(3):2580–9. doi: 10.1002/cam4.5118 (PMC9939223; doi:10.1002/cam4.5118)
Supplement: Supplementary file 1 — Appendix [file CAM4-12-2580-s001.zip › CAM4_5118_S3_Model_Code_SuppInfo.docx]

**NONMEM code of the final model**

$PROBLEM 5FU PK model

$INPUT

ID
SMIB ; SMI of the back muscle
EVID
BSA
TIME
RATE
DV
AMT
MDV

$DATA …

$SUBROUTINES ADVAN1 TRANS2

$PK

CLCOV = (1 +THETA(4) * (BSA - 1.97))*EXP(THETA(5)*(SMIB-3.78)) ; Covariate relations

TVCL = THETA(1) * CLCOV

CL = TVCL * EXP(ETA(1))

TVV=THETA(2)

V = TVV * EXP(ETA(2))

S1 = V

$ERROR

IPRED = F

DEL = 0

W =SQRT((THETA(3) * IPRED)**2)

Y = IPRED + W * EPS(1)

IF(W.EQ.0) DEL = 0.0001

IRES = DV-IPRED

IWRES = IRES/(W+DEL)

$THETA

(0, 223) ; CL

46.1 FIX ; V

(0, 0.214) ; prop. error

0.794 ; BSA on CL

0.057 ; SMIB

$OMEGA

0.0437 ; IIV CL

0.261 FIX ; IIV V

$SIGMA 1 FIX

$EST METHOD=1 INTER MAXEVAL=9999 NOABORT SIG=3 PRINT=1 POSTHOC

$COV UNCONDITIONAL SLOW MATRIX=S
