# Supplementary material for: Influence of the skeletal muscle index on pharmacokinetics and toxicity of fluorouracil
Source: Cancer Med. 2022 Aug 8;12(3):2580–9. doi: 10.1002/cam4.5118 (PMC9939223; doi:10.1002/cam4.5118)
Supplement: Supplementary file 1 — Appendix [file CAM4-12-2580-s001.zip › CAM4_5118_S4_Toxicity_Analysis_SuppInfo.docx]

**Toxicity analysis**

Tab. S4-1 Frequency of clinically relevant adverse event severity

| **Adverse event** | **Number of patients with CTCAE grade ≥2 (% of total patients)** |
| --- | --- |
| Diarrhea | 38 (34.2) |
| Stomatitis | 2 (1.8) |
| Polyneuropathy | 9 (8.1) |
| Hand-foot syndrome | 4 (3.6) |
| Fatigue | 8 (7.3) |
| Nausea | 13 (11.7) |
| Emesis | 15 (13.5) |

CTCAE: Common Terminology Criteria for Adverse Events

Tab. S4-2 Results of the logistic regression analysis

| **Skeletal muscle index** | **Adverse event** | **Odds ratio** | **95% confidence intervals** |
| --- | --- | --- | --- |
| **Psoas major** | Diarrhea | 0.793 | 0.461 – 1.46 |
|  | Stomatitis | 0.125 | 0.002 – 2.43 |
|  | Polyneuropathy | 0.513 | 0.127 – 1.75 |
|  | Hand-foot syndrome | 1.78 | 0.288 – 7.96 |
|  | Fatigue | 0.155 | 0.033 – 0.595* |
|  | Nausea | 0.657 | 0.227 – 1.71 |
|  | Emesis | 0.428 | 0.137 – 1.19 |
| **Back muscle** | Diarrhea | 0.922 | 0.706 – 1.20 |
|  | Stomatitis | 0.522 | 0.128 – 1.65 |
|  | Polyneuropathy | 0.524 | 0.287 – 0.900* |
|  | Hand-foot syndrome | 1.80 | 0.806 – 4.40 |
|  | Fatigue | 0.853 | 0.523 – 1.37 |
|  | Nausea | 0.940 | 0.617 – 1.42 |
|  | Emesis | 0.760 | 0.491 – 1.15 |
| **Total muscle (Hounsfield method)** | Diarrhea | 0.977 | 0.87 – 1.09 |
|  | Stomatitis | 0.800 | 0.386 – 1.31 |
|  | Polyneuropathy | 0.882 | 0.674 – 1.11 |
|  | Hand-foot syndrome | 1.07 | 0.751– 1.43 |
|  | Fatigue | 0.869 | 0.678 – 1.07 |
|  | Nausea | 0.901 | 0.733 – 1.08 |
|  | Emesis | 0.859 | 0.691 – 1.04 |
| **Total muscle (Segmentation method)** | Diarrhea | 0.982 | 0.948 – 1.02 |
|  | Stomatitis | 0.876 | 0.708 – 1.03 |
|  | Polyneuropathy | 1.00 | 0.937 – 1.07 |
|  | Hand-foot syndrome | 1.04 | 0.941 – 1.13 |
|  | Fatigue | 0.947 | 0.882 – 1.01 |
|  | Nausea | 0.949 | 0.892 – 1.00 |
|  | Emesis | 0.975 | 0.920 – 1.03 |

*significant at *p*<0.05
